# Estimation of Nitrogen Pools in Irrigated Potato Production on Sandy Soil Using the Model SUBSTOR

**DOI:** 10.1371/journal.pone.0117891

**Published:** 2015-01-30

**Authors:** Rishi Prasad, George J. Hochmuth, Kenneth J. Boote

**Affiliations:** 1 Soil and Water Science Department, Institute of Food and Agricultural Sciences, University of Florida, Gainesville, Florida, United States of America; 2 Agronomy Department, Institute of Food and Agricultural Sciences, University of Florida, Gainesville, Florida, United States of America; NERC Centre for Ecology & Hydrology, UNITED KINGDOM

## Abstract

Recent increases in nitrate concentrations in the Suwannee River and associated springs in northern Florida have raised concerns over the contributions of non-point sources. The Middle Suwannee River Basin (MSRB) is of special concern because of prevalent karst topography, unconfined aquifers and sandy soils which increase vulnerability of the ground water contamination from agricultural operations- a billion dollar industry in this region. Potato (*Solanum tuberosum* L.) production poses a challenge in the area due to the shallow root system of potato plants, and low water and nutrient holding capacity of the sandy soils. A four-year monitoring study for potato production on sandy soil was conducted on a commercial farm located in the MSRB to identify major nitrogen (N) loss pathways and determine their contribution to the total environmental N load, using a partial N budget approach and the potato model SUBSTOR. Model simulated environmental N loading rates were found to lie within one standard deviation of the observed values and identified leaching loss of N as the major sink representing 25 to 38% (or 85 to 138 kg ha^-1^ N) of the total input N (310 to 349 kg ha^-1^ N). The crop residues left in the field after tuber harvest represented a significant amount of N (64 to 110 kg ha^-1^N) and posed potential for indirect leaching loss of N upon their mineralization and the absence of subsequent cover crops. Typically, two months of fallow period exits between harvest of tubers and planting of the fall row crop (silage corn). The fallow period is characterized by summer rains which pose a threat to N released from rapidly mineralizing potato vines. Strategies to reduce N loading into the groundwater from potato production must focus on development and adoption of best management practices aimed on reducing direct as well as indirect N leaching losses.

## Introduction

Anthropogenic nitrogen (N) inputs are known to have significant impacts on terrestrial and aquatic ecosystem N cycles [1–2]. Trends of increasing nitrate-N concentration in surface and ground water bodies have called attention to actions necessary for improvements in water quality [3–4]. Presence of substantial nitrate amounts in water has environmental, social, and economic implications. Algal blooms in rivers and springs, dead zones in bays and seas, and risks of methemoglobinemia from high nitrate concentrations in drinking water are several of these implications [5–6].

Both point and non-point sources have been found responsible for the increasing concentrations of nitrate in water bodies. Agriculture is considered a major contributor to elevated nitrate concentration in water bodies via leaching and runoff [6–8]. Nitrate leaching is of special concern in Florida because of impairment of springs and rivers valued for their recreational and aesthetic value drawing millions of tourists every year. As of January 2010, the Florida Department of Environment Protection (FDEP) identified 14 springs and 10 water bodies, deriving their flow from ground water, as impaired, due to excess nitrate-N [9].

The concern over nitrate pollution is acute for the Middle Suwannee River Basin (MSRB) in northern Florida, where land features are characterized by unconfined aquifers, karst systems with sinkholes, springs, solution conduits, and highly permeable sands overlying the upper Floridian aquifers allowing the opportunity for direct hydraulic and geochemical interactions between surface water and groundwater [10]. Agriculture is a billion dollar industry in this region. Nitrate leaching from agricultural regions to groundwater and groundwater fed rivers (via interior-drained karst areas) such as the Suwannee River and springs in the basin have shown an increasing trends in nitrate-N concentrations from 1971 to 2006 [6, 11, 12]. The river’s baseline annual median NO_3_-N concentration of 0.50 mg L^-1^ N in 1979 increased to 0.72 mg L^-1^ N by 2005. Springs in the basin have nitrate-N concentrations between 0.1 and 10 mg L^-1^ (http://www.mysuwanneeriver.org/nitrates.htm). The contribution of this basin in annual NO_3_-N loading to the Gulf of Mexico has been reported to have increased from 3500 Mg yr^-1^ N in 1979 to 6200 Mg yr^-1^ N in 2005, a 75% increase in N loading rate over a period of 25 water years [12].

Potato is among several irrigated vegetable crops grown in the MSRB. The potato crop is targeted for this study because of the potential for high N losses from such a production system due to the shallow root system of the potato plant [13] and sandy nature of Florida soils having poor water and nutrient holding capacity and little organic matter [14]. Further, the growers in the region sometimes apply more than the University of Florida recommended N fertilizer rate (currently 224 kg ha^-1^ N; [15]) as an insurance against unpredictable rainfall patterns that promote fertilizer losses.

The idea of implementing best management practices (BMPs) for N fertilization in potato production requires a good understanding of the balance between N inputs and outputs for the potato crop. Several studies (mainly dairies) have documented farm nutrient balances [16–19], however nutrient balances for crops grown in Florida’s sandy soils, especially potatoes, have not been studied extensively. A nutrient budget is prepared by accounting for all the inputs and outputs of the chosen nutrient for a defined cropping system over a defined period of time [20]. Nitrogen budgets for crop-soil system can serve not only as a metric for evaluating the N flow in the system but can also help determine the N loading in the environment. A crop-soil system N budget requires quantification of all the sources (inputs) and sinks (outputs) of N for the given system. Accurate measurement of several components of the N budget (such as volatilization, denitrification, and atmospheric deposition) is challenging due to spatial and temporal variations in soil N fluxes and poses analytical limitations. Further, the uncertainty in measurement of individual inputs or outputs determines the overall uncertainty of the budget [21] and may lead to confusion and wrong conclusions [20]. Often, a partial N budget is used to describe input-output balances. The budget is considered as partial because losses from soil erosion, surface runoff losses, and gaseous losses from the soil are not considered, nor are additions from atmospheric deposition, sediment deposition, or collection of runoff from other areas. For example, an N budget created by the National Research Council [22] did not consider N inputs such as wet and dry deposition, N in planted seeds, non‐symbiotic N fixation and foliar absorption of atmospheric N. With advancement in computing technology, crop models have emerged as an important decision support tool. Monteith [23], defined a crop model as a “quantitative scheme for predicting the growth, development and yield of a crop, given a set of genetic coefficients and relevant environmental variables”. Decision Support System for Agrotechnology Transfer (DSSAT) is a popular simulation model that contains a suite of crop models [24–25]. The current software application program of DSSAT (v4.5) is comprised of over 28 crop simulation models. The Simulation of Underground Bulking Storage Organs (SUBSTOR) subroutine is among several crop models included in the DSSAT that can simulate potato growth, development, and yield [26]. The model also incorporates N transformation, transport, and uptake along with simulation of yield and N balance which can serve as an important guide in crop management decisions.

There is a lack of information in literature regarding environmental N loading rates from cultivation of potato on a commercial setting in an environmentally sensitive area comprising of sandy soils and karst features which makes the ground and surface water susceptible to nitrate-N contamination. Nevertheless, quantifying all the possible N loss pathways presents methodological challenges, especially in a commercial setting in contrast to controlled research scale plots. This study presents a method of estimating N loading rates using potato model SUBSTOR and provide significant insights into the N cycling in such agricultural settings. This research will also serve as an example to the farmers and the scientific community in general to look for ways to increase N budget efficiency by evaluating N budget components of potato production in an environmentally sensitive area prone to N losses. In addition, environmental regulators will have accurate data on which to base cost-share funding programs to assist farmers in focusing on the appropriate BMPs to reduce losses of N from the farms.

The farm chosen for this study is of special concern due to its close proximity to the Suwannee River (the river is located 1.5 km south west of the farm) where maximum N load in parts of the river were identified by Hornsby [12] and Pittman et al. [27]. A study carried by Albert [28] at the farm calibrated the SUBSTOR model using one season of data and identified the need to verify or evaluate the calibrated model using multiple seasons of independent data. Model evaluation involves a comparison between independent field measurements and outputs created by the model. This study provides an independent evaluation of the calibrated model [28] by using data collected during four growing seasons (2010–2014) at several locations in the 2020 ha farm. The parameters selected for model evaluation were N concentrations in shoot, root and tuber, plant N uptake, tuber dry matter yield, above-ground dry matter, fresh tuber yield and environmental N loading rates. Evaluation of soil water and N transport during the potato growing season was presented by Albert [28].

The objectives of this study were (i) to perform the evaluation of a previously calibrated SUBSTOR potato model using four seasons of independent data collected at several locations on a 2020 ha commercial farm located in the MSRB in northern Florida. (ii) to use the evaluated model to identify the major N loss pathway from potato production in the MSRB and provide model generated estimation of the contributions of individual N loss pathways towards total environmental N loading rate in MSRB.

## Materials and Methods

### Regional Geology, and Climate of Middle Suwannee River Basin

The study site is located in the MSRB where the underlying Floridan aquifer is unconfined and agriculture is the dominant land use. The soils in the agricultural areas belong primarily to Entisols or Ultisols where texture in the root zone is usually sand or fine sand regardless of the soil order [29]. The study site lies along a physiographic region known as the Cody Escarpment which is marked by thick sands and eroded Hawthorne formation consisting of a thin and pocketed clay to absence of clay mantle atop the limestone [30]. A Ground Penetrating Radar (GPR) study carried at the site indicated wide variability in clay depths (A variogram indicated a variance of 0.5 m^2^, a spatial correlation of 200 m and a nugget of 0.175 m^2^, [28]). These geological features (Cody Escarpment, eroded Hawthorne formation) increase the threats to water quality of Suwannee River. Additionally, the sandy soils in this region are susceptible to drought, making irrigation critical for the economic viability of the farming operation.

The climate of the Suwannee River Basin is a mixture of warm temperate and subtropical conditions [31]. Based on the long term National Oceanic and Atmospheric Administration climate data (1900–2003), the mean annual temperature was 20.3°C with July as the hottest month (maximum mean monthly temperature of 27.3°C) and January as the coldest month (minimum mean monthly temperature of 12.3°C) [31]. Annual precipitation averaged about 1356 mm with a pronounced wet season in the summer months (June through September). The mean annual ET was 1036 mm with largest mean monthly value of 132 mm in June and a minimum of 33 mm in December [31]. Summer rainfalls are mostly associated with thunderstorm, hurricanes, or tropical storms while winter rainfalls occur due to mid-latitude frontal weather systems.

### Study Site and Soil Characteristics

**Fig 1:**
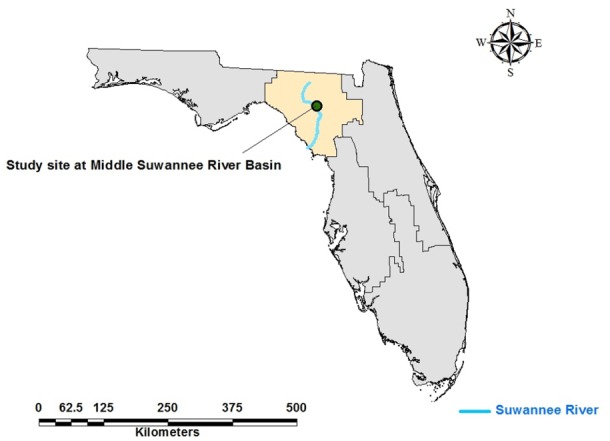
Study farm located in Middle Suwannee River Basin, Florida. The Suwannee River (highlighted) is 1.2 km south-west to the farm. The farm has 42 individual center pivots (or fields) of average size 55 ha.

The study site is a commercial farm located in O’Brien, Suwannee County, Florida, near latitude 30.04 and longitude-82.94 (Fig. 1). This study was conducted at the request of the farm owner and written permission was obtained prior to the field work. No permits or approvals from any state or federal regulatory agency were required to carry out this study. The farmer was interested in improving nutrient management as part of adopting BMPs and requested more information on the potential N losses from current production practices. The diversity of agricultural enterprises on this farm and its proximity to the Suwannee River make this farm an important field laboratory to study N losses from the agricultural systems. An earlier study carried out by Hornsby [12] and Pittman et al. [27] indicated an occurrence of maximum N load in parts of the river near the farm. There are several other small farms located in close proximity to this farm. The subject farm for this study is a large, diversified vegetable, row crop, and cattle feeding operation with total land area of 2020 ha. Main crops grown on the farm are potato (Solanum tuberosum L.), sweet corn (Zea mays var. saccharata), silage corn (Zea mays var. indentata), peanut (Arachis hypogaea), and cotton (Gossypium hirsutum), in addition to several other vegetable crops. The crops are irrigated by 42 individual center pivot irrigation systems with an average pivot (or field) size of 55 ha. A center pivot irrigation system is a form of overhead sprinkler irrigation in which a circular area centered on the pivot is irrigated. The source of water for the pivot system was groundwater drawn from an average depth of 22 m below the surface (personal communication from farmer). The average daily irrigation rate was 8 mm d^-1^ and application decisions were made based on crop growth stage and soil moisture conditions. The farmer maintained the soil moisture approximately near field capacity (personal communication from farmer) to prevent water stress in plants. The word “field” and “pivots” are used interchangeably hereafter.

In a previous study in one of the fields (Pivot 12) at the farm, Albert [28] reported the land surface elevation ranged from 13.7 to 15.3 m above mean sea level (msl) whereas the average elevation of the top of the Floridan Aquifer fluctuated between 7.3 m (annual low) to 8.5 m (annual high) above msl. Also, the average depth to limestone from the surface was 7 m. The clay layer at the site was semi-continuous at depths between 0.6 to 8 m below the soil surface. A potentiometric surface map of the farm documented a southwesterly flow of groundwater from the farm toward the Suwannee River.

Soils in the study fields are classified as Alpin fine sand (Thermic, coated lamellic Quartzipsamments) [32] belonging primarily to hydrologic group A. As reported by Albert [28], the volumetric water content of the soil was 6 to 7% at field capacity and 2% at wilting point, saturated hydraulic conductivities of 19 cm h^-1^ and 16 cm h^-1^ for 0 to 0.5 m and 0.50–1.0 m depths, respectively, and average bulk densities of 1.48 g/cm^3^ and 1.56 g/cm^3^ for 0 to 0.50 m depths and 0.50 to1.0 m depths, respectively. Soil sampling conducted during the current study period (2010 to 2013) indicated an average soil pH of 6.5 (1:2 soil-solution ratio measured in water), and tested high in Mehlich1-P (> 31–60 ppm) according to Mehlich-1 P index for vegetable production in Florida [15]. The average soil organic N concentration was 0.03% in upper 0.3 m soil layer and the average organic carbon concentrations were 0.32%, 0.12%, 0.05% in 0 to 0.3 m, 0.3 to 0.6 m, and 0.6 to1.0 m depths, respectively.

### Cultural Practices of the Irrigated-Potato Production System

The study fields were managed by the cooperating farmer including crop selection, irrigation management, nutrient management, among other production aspects (e.g. pest control etc.). Information on crop management practices are presented in Table 1. The potato seed pieces of the cultivar ‘Red La Soda’ were planted in raised beds (The beds were hill shaped in single row systems (consecutive beds separated 1.01 m apart) with a peaked top and side slopes ending at the furrow position) between late January to end of February and plants were desiccated with a foliar acting herbicide (diquat at 1.2 l ha^-1^) in preparation for harvesting in late May. The seasonal fertilizer applications comprised of 4 to 5 split-applications of N, 2 split-applications of P (one as pre-plant application and the other at planting) and 2 split-applications of K (one as pre-plant application and the other at emergence). The pre-plant applications of NPK were made through a ground spreader whereas “at-plant” application of N and P was made through the planter. The third and fourth applications of N were made by a liquid fertilizer applicator that applied the fertilizer in a band on the side of the bed. The fifth split-application of N was optional, necessitated by heavy rains (a rainfall of 76 mm in 3 days or 102 mm in 7 days) and made through the center pivot sprinklers. Irrigation was managed depending on weather conditions, crop growth stage, and soil moisture status. The total seasonal water applications made through center-pivot sprinklers ranged between 312 to 388 mm.

**Table 1:** Crop management information for potato production at the study farm in the MSRB during the model evaluation period (2010–2013).

Year	——2010———	—————-2011————————		—————————2012————————————		——-2013——-
Field identification	Pivot 19	Pivot 12	Pivot 17	Pivot 10	Pivot 18	Pivot 12
Description						
Planting date	02–10–2010	01–28–2011	2–12–2011	1–31–2012	2–19–2012	2–14–2013
Row spacing (m)	1.01	1.01	1.01	1.01	1.01	1.01
Plant spacing (m)	0.10	0.10	0.10	0.10	0.10	0.10
Planting depth (m)	0.16	0.16	0.16	0.16	0.16	0.16
Previous crop	Sorghum (harvested Nov 2009)	Corn (harvested Nov 2010)	Corn (harvested Nov 2010)	Cotton (harvested Nov 2011)	Corn (N half) + Cotton (S half) (harvested Nov 2011)	Cotton (harvested Nov 2012)
Cultivar	Red LaSoda	Red LaSoda	Red LaSoda	Red LaSoda	Red LaSoda	Red LaSoda
Harvest date(Harvested before desiccation)	5/20/2010	4/28/2011	5/20/2011	5/2/2012	5/20/2012	5/20/2013
Total Irrigation (mm)	312	330	324	388	381	319
Total N (kg ha^-1^)	265	278	285	285	248	248
Total P (kg ha^-1^)	47	51	51	49	49	49
Total K (kg ha^-1^)	380	354	358	339	337	343

The potato crop was grown in spring seasons each year at several locations on the study farm identified by pivot (or field) numbers.

### Field Methods

Field methods for this study focused on collection of plant and soil samples for four successive growing seasons (spring 2010-spring 2013) at several locations in the study site. The crop was monitored for biomass accumulation and N uptake each spring season near plant harvest. Soil cores were used to determine soil mineral-N concentrations. Different fields were sampled each year following the grower’s crop rotation program. The fields selected for the study were identified as Pivot-19 in 2010; Pivots-12 and-17 in 2011; Pivots-10 and 18 in 2012 and pivot-12 in 2013. To adequately represent the conditions within each field, plants and soil were sampled at twelve random locations within the 55 ha fields. Each location within the field was considered as one observational unit. All plants in a 1.5-m length of row at each site were harvested one week prior to application of plant desiccant in preparation for commercial harvesting. The plants were separated into tubers, shoots (comprised of senescent leaves, fresh leaves, stems and stolons), and roots. The tubers were graded into USDA sizes A, B and C [33] and fresh weights were recorded to determine the fresh tuber yield. Soils were sampled in the center of the potato plant beds (duplicate samples composited at each observational unit) using a soil probe (diameter 0.05 m) to a depth of 0.3 m in two increments, 0 to 0.15 m and 0.15 to 0.30 m. Any inorganic N beyond 0.3 m soil profile was considered lost since 90% of the potato roots were in the upper 0.25 m of the soil profile where active water and nutrient uptake occur [34–35].

### Laboratory Methods

The individual plant parts were oven dried at 70°C for 48 to72 hours until constant dry weight was measured and values were recorded. The dried plant parts were ground in a Wiley mill to pass a 2 mm screen, and mixed well. Nitrogen determinations were made on the tissue samples using the Kjeldahl digestion followed by semi-automated colorimetry (EPA Method 351.2) using Technicon AAII (Technicon Instruments Corp., Tarrytown, NY, USA). Tissue nitrate-N was determined using 2N KCl extraction followed by semi-automated colorimetry (EPA Method 353.2). The average (± standard deviation) nitrate-N concentration was found to be 0.16 ± 0.10% for shoot and negligible (below method detection limit) for tuber samples. These values represented negligible amounts of tissue nitrate-N present at crop maturity. Therefore, all plant tissue N determined following Kjeldahl digestion was considered as total N.

The soil cores were air dried (40°C for 48 hours) and the soil samples were analyzed for Kjeldahl- N (TKN) and 1 M KCl extractable nitrate and ammoniacal-N according to the standard procedures [36]. Soil TKN was determined following Kjeldahl digestion and analysis of N by automated colorimetric analysis (EPA Method 351.2) using the Alpkem Flow Solution IV (OI Analytical, College Station, TX, USA). Nitrate-N and ammoniacal-N determinations were made by automated colorimetric analysis (EPA method 353.2 and 350.1(modified) respectively) using the Alpkem Flow Solution IV (OI Analytical, College Station, TX, USA). Organic N was calculated as the difference between Kjeldahl-N and ammoniacal-N. Organic carbon was determined by the wet digestion method [37].

All conversions (or upscaling of data to a hectare basis) relevant to the study (such as crop N uptake, dry matter accumulation, soil initial and final mineral N and other parameters) were made according to Prasad and Hochmuth [38].

### Calculation of Nitrogen Mass Balance

Nitrogen mass balance for each growing season was calculated by quantifying the sources (or inputs) and sinks (or outputs) of N for potato crop using a partial N budget approach (see Equation 1). Nitrogen input components that had higher uncertainty associated with their measurement were not accounted in the budget and justifications for doing so were addressed as indicated by Oenema et al.[20] and Meisinger and Randall [21]. Four N inputs were not included in the budget: 1) seed-N, 2) N contributions from net mineralization (mineralization-immobilization) of crop residues and soil organic matter, 3) N contributions from irrigation water and, 4) N contribution from atmospheric deposition. Nitrogen in tuber seed represented a minor amount (3 to 4 kg ha^-1^) in the budget and was not included. Nitrogen contributions from net mineralization of soil organic matter and plant residues were not included in budget due to high uncertainty associated with their measurements. Also, the fate of N resulting from mineralization of plant residue was not clear in Florida sandy soils. In a study by Bundy and Andraski [39] on irrigated sandy soils in Central Sand Regions of Wisconsin, N mineralized from crop residues was not recovered in the subsequent crop and were lost via leaching. Nitrogen present in irrigation water was not included in the budget due to presence of seasonal fluctuations in water nitrate-N concentration. Water sampling from two irrigation wells during the study period showed low nitrate-N concentrations during spring season (less than 1 mg L^-1^) (potato growing season) and peaked during fall season (20 mg L^-1^) when potatoes are not grown. Similar observation of seasonal nitrate-N fluctuations in groundwater was also reported by Albert [28]. He reported elevated nitrate-N concentrations in groundwater began to appear in July (after the harvest of potato crop) and peaked in October, 2000 at 35 mg L^-1^. The contribution of nitrate-N from irrigation water was found to range between 1 and 3 kg ha^-1^ season^-1^ and was not included in the budget. Contribution of wet and dry atmospheric N deposition was minor (1 to 2 kg ha^-1^ season^-1^N) relative to other N inputs and not included in the budget. Wet N deposition estimate was based on the precipitation amounts, length of the growing season, and ion concentrations obtained from a nearby National Atmospheric Deposition Program monitoring station located at Branford site, Florida (FL03) (NADP; available at http://nadp.sws.uiuc.edu/). Dry deposition contributed 5% of the wet deposition. The estimate on dry deposition was obtained from Clean Air Status and Trends Network (CASTNET) site located at Sumatra, Florida (site ID: SUM156) (CASTNET; available at http://www.epa.gov/castnet/javaweb/site_pages/SUM156.html).

The difference between the input and output of N budget was considered as unaccounted-for N and used as a metric for estimation of seasonal environmental N loading rate. The unaccounted-for N was comprised of leaching loss and gaseous loss via volatilization and denitrification pathways. Surface runoff loss was not observed on study fields, hence it was not accounted for in the budget. Following mass balance equation was used in estimation of environmental N loading rate:

$$N_{env\;load.}=N_{In.}+N_{fert}-N_{crop}-N_{Fi.}$$ 1

Where, N_*env load*_ is environmental N loading (or unaccounted-for N), N_*in*._ is initial mineral N in soil (0.3m) before planting, N_*fert*._ is the N from fertilizer application, N_*crop*_ is the crop N uptake, N_*Fi*._ is the mineral N present in soil (0.3m) at crop harvest.

### DSSAT-SUBSTOR Potato Model

The SUBSTOR model has been tested extensively by several researchers for its broader applications such as yield predictions, N and irrigation response relationships with yield, and impact of climate change on potato production [40–44]. The DSSAT also has the capability to simulate changes in soil water, carbon and N that occur during crop development phase. A detailed description of the hydrological component of SUBSTOR can be found elsewhere [45–46]. The DSSAT shell allows the user to input, store, and output information for crop simulations, sensitivity analyses, model calibrations, and model evaluation. All the crop models in DSSAT use common nutrient transport and hydrologic routines and differ only in the methods used for plant growth. For this study, specific methods used within DSSAT were: Ritchie’s method for soil-water transport and infiltration (tipping bucket), Priestly-Taylor/Ritchie method for evapotranspiration, daily canopy curve for photosynthesis, century model for soil organic matter simulations, and Suleiman-Ritchie method for soil evaporation [25].

The minimum data set required to run the model included weather data, soil data, genetic coefficients to define a cultivar’s unique characteristics, and information on crop management practices. A flow diagram of the application and processes for DSSAT can be found in Jones et al. [25]. The model took into account several processes simultaneously and each simulation run consisted of calculations of the phenological development, formation of leaf, stem and root biomass and its partitioning, available soil water and its utilization by the crop, and the N balance and its distribution to crop organs [43].

### Model Inputs

**Soil Input Data**. Soil input data consisted of estimates of soil parameters such as soil texture, soil pH, bulk density, drained upper limit (DUL) (corresponds to soil moisture content at water potentials in the range of-10 to-33 kPa (similar to field capacity)), drained lower limit or lower limit of plant-extractable soil water (DLL) (similar to wilting point), hydraulic conductivity, and organic carbon. The estimates were obtained partly from the work of Albert [28] who used the same farm site to conduct his research, as well as through the field measurements (soil pH, organic carbon, and bulk density) conducted during the study period (2010 to 2013). Important soil properties are presented in Table 2.

**Table 2:** Soil characteristics at the study farm in MSRB.

Soil layer	Silt†	Clay†	Coarse fraction	Organic C	Bulk density	LL	DUL	SAT	K_sat_	SRGF
m	———————————————%————————————-				Mg m^-3^	—————-% vol————————			—cm h^-1^—	—0–1—
0–0.15	2.5	1.7	1.4	0.32	1.48	0.017	0.097	0.388	19	0.75
0.15–0.30	3.7	1.2	1.1	0.32	1.48	0.017	0.097	0.388	19	0.5
0.3–0.60	3.9	1	1.4	0.12	1.56	0.019	0.08	0.388	16	0.15
0.6–1.0	3.9	1	1.4	0.05	1.56	0.019	0.08	0.388	16	0.15

Physical meaning of the abbreviations used in the table are: Silt, clay and coarse fragment content, organic carbon (C), bulk density, water content at wilting point (LL), drained soil water limit (DUL), water content at saturation (SAT), hydraulic conductivity(K_sat_) and soil rooting preference function (SRGF).

†Silt, Clay, and Sand percentage sum to 100. Coarse fragment is non-water holding

**Weather Data**. The observed daily weather data for the research site were obtained from the satellite data through NASA POWER (NASA Prediction of Worldwide Energy Resource) on a spatial resolution of 1° latitude by 1° longitude grid. (http://power.larc.nasa.gov/cgibin/cgiwrap/solar/agro.cgi?email=agroclim@larc.nasa.gov). The data consisted of daily maximum temperature (T_max_), minimum temperature (T_min_), and solar radiation (S_rad_) for the period 2010 to 2013. The daily rainfall data for the study site were obtained from the Suwannee River Water Management District (SRWMD) (http://www.srwmd.state.fl.us/index.aspx?NID=345). Weather information for the study period is presented in Table 3.

**Table 3:** Mean monthly solar radiation, maximum and minimum temperatures, and monthly total rainfall at the study farm for spring growing seasons 2010 to 2013.

	Solar Radiation (MJ m^-2^ d^-1^)				Maximum Air Temperature (°C)				Minimum Air Temperature (°C)				Rainfall (mm)			
Month	2010	2011	2012	2013	2010	2011	2012	2013	2010	2011	2012	2013	2010	2011	2012	2013
Jan.	11.9	10.9	12.3	11.2	13.0	14.5	20.2	21.1	3.7	4.2	9.2	9.7	178.1	153.7	43.7	17.3
Feb.	13.8	14.1	10.9	12.1	13.9	18.7	21.2	20.3	3.8	8.7	11.1	8.4	90.4	98.3	59.9	175.5
Mar.	17.0	17.7	17.8	18.9	18.6	24.0	27.2	19.8	8.2	12.8	15.6	6.6	83.1	104.4	102.9	83.3
Apr.	22.4	23.3	22.8	19.4	25.5	28.9	29.1	26.3	14.6	16.1	16.9	14.4	67.1	116.1	11.9	87.4
May.	22.2	25.6	23.3	23.3	30.3	32.0	31.8	29.4	20.9	19.0	20.9	17.1	229.1	22.6	245.1	34.5
Jun.	23.0	24.3	21.0	20.1	31.1	32.7	30.7	31.7	23.9	23.9	22.3	23.1	174.5	92.7	695.2	199.1

**Cultivar Genetic Coefficients**. The cultivar ‘Red La Soda’ was grown during the study period in all fields. Five genetic coefficients (G2, G3, PD, P2, and TC) are required to characterize the potato crop growth and development. Briefly, they are the leaf expansion rate (G2), tuber growth rate (G3), determinacy (PD), upper critical temperature sensitivity of tuberization (TC), and sensitivity of tuber initiation to long photoperiod (P2). These coefficients were determined and evaluated by Albert [28] at the same study site for the cultivar ‘Red La Soda’. He reported the values as 2000, 22, 0.7, 0.4, and 19 for G2, G3, PD, P2, and TC, respectively. P2, TC and PD are unitless coefficients whereas G2 has units of cm^2^ m^-2^ d^-1^ and G3 has units of g m^-2^ d^-1^. These coefficients were adopted for model evaluation using the data collected during the 2010–2013 study period.

**Crop Management Data**. Crop management data for the study period such as row spacing, planting depth, planting date, emergence date, planting method, and irrigation and fertilizer management were all obtained from the cooperating farmer and presented in Table 1. We assumed a 10% loss of water during irrigation applications hence an efficiency factor of 0.9 was used in DSSAT for calculation of effective irrigation for the growing seasons. Contribution of nitrate-N from irrigation water was not accounted for in the model run because of the seasonal fluctuations in nitrate-N concentrations in irrigation water observed during the study period. Each potato crop received four split-applications of inorganic N fertilizers during the growing season, totaling between 248 to 285 kg ha^-1^. The N fertilizers used were monoammonium phosphate (MAP) and urea ammonium nitrate (UAN). The MAP was applied pre-plant and at planting whereas UAN was applied as side-dressing. Sometimes a fifth application of ammonium nitrate sulfate was made through the pivot irrigation system after a heavy rainfall event (A heavy rainfall event for potato production in north Florida region is defined as a rainfall of 76 mm in 3 days or 102 mm in 7 days [47]).

### Model Calibration and Evaluation

Albert [28] used one season of plant and soil data to calibrate the model (using DSSAT v3.5) and identified the need to evaluate the calibrated model using multiple seasons of data. The present study focused mainly on model evaluation (using DSSAT v4.5.1.023) for N concentrations in shoot, root, and tuber, plant N uptake, tuber dry matter yield, above-ground dry matter (shoots), fresh tuber yield and environmental N loading rates using four seasons of data collected at multiple locations on the farm. Model evaluations for water and N transport in soil profile were not carried out in the present study and can found in the work of Albert [28]. We assumed that the most sensitive parameters (saturation and field capacity; [48] required for calculation of soil water balance did not change over short period of time and N removal by plants represented a major portion in the overall N budget. Hence more focus was given to evaluation of plant N uptake and yield. For our purpose, the values of soil parameters such as DUL, DLL, Ksat, etc. (Table 2) were adopted from work of Albert [28] to simulate water and N transport through the soil profile (for the same soil as used for the current study). An additional modification to the calibrated model included initialization of soil organic matter pools through use of the CENTURY model to simulate N mineralization [49–50]. The soil organic C pools were initialized by providing the values of two fresh organic matter (FOM1 and FOM2) pools and three soil humic organic matter (SOM1, SOM2 and SOM3) pools. The model initializes the soil organic C by subtracting the supplied FOM (previous crop residue) from the total measured organic C to obtain the humic SOM. After that, the model assumes the fractions of SOM1 and SOM2 to be 5% and 95% of the remaining amount of SOM, respectively [50]. The SOM3 value was estimated according to Basso et al [49]. For this study, the total SOC and estimated SOM3 were 0.32% and 0.3% for the 0 to 0.15 m soil layer, 0.12% and 0.1% for 0.15 to 0.3 m soil layer, 0.08% and 0.07% for 0.30 to 0.60 m soil layer and 0.05% and 0.04% for 0.60 to1.0 m soil layer, respectively.

The initial conditions of the soil profile at the beginning of the simulation was set in the soil files for each pivot. The simulation start date was set at 15 November, close to the date when the farmer harvested the fall row crop (information on previous crop is presented in Table 1). The period between harvest of fall row crop and planting of spring potato was kept as fallow in all simulations for all pivots during the study period. The initial soil water content, organic N, ammonium, and nitrate-N concentrations, were set based on the estimates of soil sampling performed during fall season at harvest. Initial crop residue was assigned an estimated value of 1500 kg/ha with residue N concentration of 1% and incorporated 100% at 0.15 m depth. Model runs with DSSAT-SUBSTOR-potato 4.5.1.023 were performed on a daily time step with the N and water balance simulations options turned “on”. Finally, model evaluations were performed on plant N accumulation and dry matter production at plant maturity to verify the reliability of the calibrated SUBSTOR-Potato model by comparing the simulation output to measured values.

Performance of model simulations (goodness-of-fit) and its accuracy in prediction were evaluated using statistical indicators of root mean square error (RMSE), and the Wilmot index of agreement (d value) [51–52] The root mean square error (RMSE) [53] between observed and simulated values and d-index [54] were computed as:

$$\text{RMSE}={\left[{\text{N}}^{-1}\sum_{\text{i}=1}^{\text{n}}{\left({\text{P}}_{\text{i}}-{\text{O}}_{\text{i}}\right)}^{2}\right]}^{0.5}$$ 2

$$d=1-\frac{\sum_{i=1}^{n}{\left(P_{i}-O_{i}\right)}^{2}}{\sum_{i=1}^{n}{\left(\left|{P^{\prime }}_{i}\right|+\left|O{'}_{i}\right|\right)}^{2}}$$ 3

Where, Pi and Oi are the predicted (or simulated) and observed values, respectively, n is the number of observations.and P’i = Pi—Oav (average of the observed data) and O’i = Oi—Oav.

Both RMSE and Willmott d-index help in evaluating the simulation capability of the model better than a correlation coefficient (r or r^2^) or 1:1 line [52]. Lower RMSE and a higher d value (close to 1.0) indicate better agreement between the model simulations and the observed data. A d value of zero indicates no predictability [52].

## Results and Discussion

### Performance of SUBSTOR Model for Nitrogen Simulations

**Fig 2:**
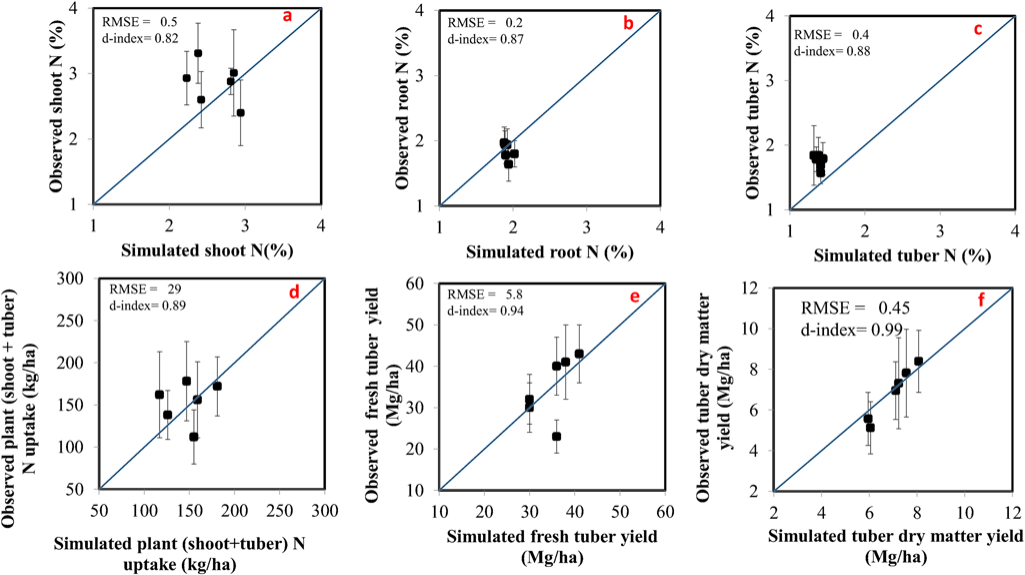
Comparisons between model simulated and observed values. (a) shoot N concentration (b) root N concentration (c) tuber N concentration (d) plant (shoot + tuber) N uptake (e) fresh tuber yield (f) tuber dry matter yield, at harvest maturity during model evaluation period (2010–2013). Error bars represent one standard deviation about the average measured value. Solid line represents (1:1 line).

**Fig 3:**
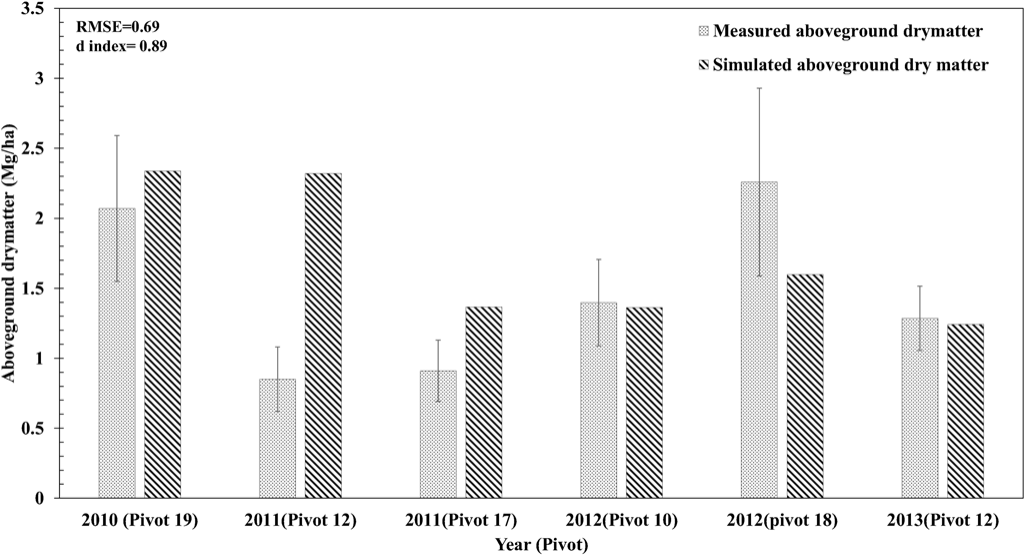
Comparisons between model simulated and observed value for aboveground dry matter accumulation for potato at harvest maturity during model evaluation period (2010–2013) at several locations (pivots) in the study farm. Error bars represent one standard deviation about the average measured value.

**Comparison of Simulated with Measured Data for Plant Parameters**. The model simulated value was found to lie within one standard deviation of the observed values. The comparisons between model simulated and observed values suggested a close agreement supported by statistical indices (RMSE and Willmott d-index) used to evaluate the accuracy of the model (Figs. 2 and 3). The model-simulated values and observed values of the root, shoot, and tuber N concentrations were found acceptable with a high d (0.87 for root, 0.82 for shoot and 0.88 for tubers) and low RMSE values (0.2% for root, 0.5% for shoot and 0.4% for tubers) (Fig. 2a, 2b, 2c). Plant (shoot + tubers) N uptake was closely simulated by the model with a d value of 0.89 and RMSE of 29 kg ha^-1^ (Fig. 2d). Dry tuber yields showed a good agreement between model simulated and observed values with a d value of 0.99 and RMSE of 450 kg ha^-1^ (or 0.45 Mg ha^-1^) (Fig. 2 f). However the model over estimated aboveground dry matter for fields-12 and-17 during 2011growing season (Fig. 3). The aboveground dry matter from other fields and sampling years were in good agreement with model simulated values (Fig. 3). The fact that the plants were harvested near maturity may have increased the loss of senescent leaves; hence the measured values were lower than the model simulated values.

Fresh tuber yield also showed good agreement between model simulated and observed values as indicated by high d-value (0.94) and low RMSE (5.8 Mg ha^-1^) (Fig. 2e). During the 2010 growing season, the plants suffered from late blight disease and the yield was reduced resulting in inflated RMSE values. The model simulated tuber dry matter yield did not show large deviations from observed values (Fig. 2f). The model lacks the capability to account for yield losses due to disease and hence over-estimated the yield during the 2010 growing season.

**Fig 4:**
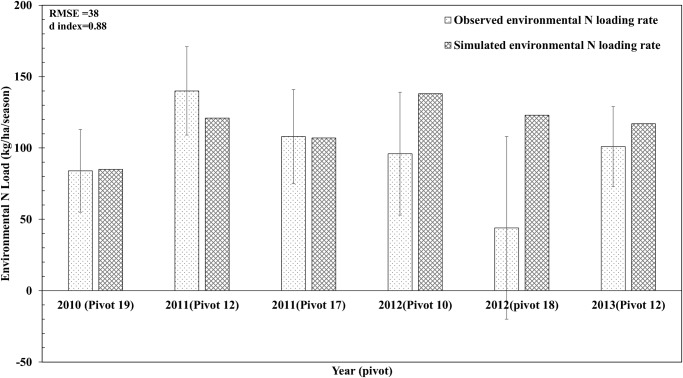
Comparisons between model simulated and observed values for environmental nitrogen loading rates from potato production at several locations (pivots) in the study farm during model evaluation period (2010–2013). Error bars represent one standard deviation about the average measured value.

**Fig 5:**
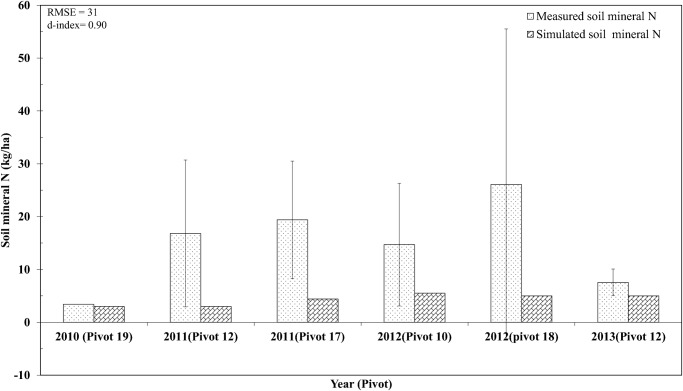
Comparisons between model simulated and observed values for soil mineral N (nitrate plus ammonium-N) left at harvest maturity in 0–30 cm soil depth at several locations (pivots) in the study farm during model evaluation period (2010–2013). Error bars represent one standard deviation about the average measured value.

**Comparison of Simulated and Measured Data for Environmental Nitrogen Loading Rates**. Unaccounted-for N in the N budget was used to approximate environmental N loading rates and comprised chiefly of leaching loss and gaseous loss of N (via volatilization and denitrification). Model simulated environmental N loading rates for most fields were in good agreement with the observed values (Fig. 4), as indicated by d-value of 0.88 and RMSE value of 37 kg ha^-1^ season^-1^. The environmental N loading rate for field-18 in year 2012 suffered from high variability and was not fully captured by the model (although model generated N budget (Table 4) showed that high amounts of mineral N was left in the soil after tuber harvest). Examination of outliers in the observed data set revealed high soil mineral N concentrations (Fig. 5). These high soil mineral N concentrations might have resulted from late application of large amounts of N (105 kg ha^-1^) in the last split (4^th^ split) that were not utilized by potato plants. During the tuber bulking phase, the potato plant slows down N uptake and starts N translocation from leaves to tubers. The presence of large amount of mineral N might have created hot spot area in the potato beds where soil sampling was carried out. This imparted greater variability to the environmental N loading rate in field-18 during 2012 season.

**Table 4:** Model simulated seasonal N budgets for potato production at the study farm during the evaluation periods (2010–2013). Nitrogen budgets were prepared for individual fields (pivots) sampled at the study farm.

Year	2010	———-2011———		———2012——-		2013
Locations	Pivot 19	Pivot 12	Pivot 17	Pivot 10	Pivot 18	Pivot 12
	————————————-kg ha^-1^ season^-1^————————————————-					
Description						
Sources of nitrogen						
1. Potential N mineralization	46	43	48	48	51	46
2. Soil mineral N before planting (1m soil profile)	16	16	16	16	16	16
3. Fertilizer N	265	278	285	285	248	248
Total	327	337	349	349	315	310
Sinks of nitrogen						
1. Crop N uptake (1a+1b+1c)	214	180	212	175	147	161
1a. Root	14	11	12	8	8	9
1b. Aboveground biomass	96	85	93	58	56	58
1c. Tuber	104	84	107	109	83	94
2. Soil mineral N after crop harvest (1 m soil profile)	11	20	13	22	30	17
3. Environmental N loading rates (3a+3b+3c)	85	121	107	138	123	117
3a. Leaching loss	81	118	103	133	119	114
3b. Volatilization loss	3	2	3	3	2	2
3c. Denitrification loss	1	1	1	2	2	1
4. Immobilization of N	17	16	17	14	15	15
Total	327	337	349	349	315	310

**Model Simulated Seasonal Nitrogen Mass Budgets for the Evaluation Periods**. The model evaluation using the data collected during the study (or evaluation period 2010–2013) indicated that the SUBSTOR-potato model adequately simulated N concentrations in shoot, root and tuber, plant N uptake, tuber dry matter yield, above-ground dry matter and fresh tuber yield. The model was evaluated for drainage and soil water nitrate concentrations by Albert [28]. Upon satisfactory evaluation of the model, the model estimates were used to construct the seasonal N budget for potato grown on individual fields. The purpose of developing the simulated seasonal N budget was to identify the major N loss pathways and quantify their contribution towards the environmental N loading rates so that BMPs can be targeted by the farmer to minimize those losses.

The inputs in the N budget comprised (1) N contributions from mineralization of soil organic matter and decomposition of plant residues (2) initial mineral-N present in the soil profile before planting (mineral N is defined as the sum of 1 M KCl extracted ammonium-N and nitrate-N) and (3) N applied through fertilizer. The outputs in N budget comprised (1) crop N uptake (2) mineral-N left in the soil after crop harvest (3) N lost as leaching (4) gaseous loss of N via volatilization and denitrification and (5) N immobilization by soil microbes. The budget components and their model estimates are presented in Table 4.

Of the total N input, the contribution of fertilizer N ranged between 79 to 82% (or 248 to 285 kg ha^-1^ N) followed by N from mineralization of soil organic matter and previous crop residue which contributed 13 to 16% N (or 43 to 51 kg ha^-1^ N). The initial mineral-N present in the soil profile contributed only 5% (or 16 kg ha^-1^N) towards the total N input. The average total input in the N budget was 331 kg ha^-1^ N.

The model estimations of the N outputs suggested that, of the total N input, 47 to 65% N (or 147 to 214 kg ha^-1^N) was taken up by the plants, 3 to 10% (or 11 to 30 kg ha^-1^ N) mineral-N was left in the soil after tuber harvest and 4 to 5% N (or 14 to 17 kg ha^-1^ N) was immobilized by microbes while the remaining N was associated with environmental N loading through leaching and gaseous loss pathways. The model estimated environmental N loading via leaching was 25 to 38% (or 81 to 133 kg ha^-1^ N) of the total N input whereas gaseous loss of N via volatilization and denitrification represented 1% (or 3 to 5 kg ha^-1^ N) in the total N input.

Model estimation of environmental N loading rates identified N leaching as the primary loss pathway at the study site and its contribution represented an average value of 111 kg ha^-1^ season^-1^ N. Mineral-N left in the soil after crop harvest was also susceptible to leaching loss in event of heavy rainfall during summer fallow period and hence represented a potential for environmental N loss via leaching. Further, total N exported off the farm represented 25 to 32% (or 83 to 107 kg ha^-1^ N) of the total N input whereas 19 to 34% N (or 64 to 110 kg ha^-1^ N) was left in the soil through shoots, roots, and senescent leaves for recycling to become part of soil organic matter.

Although N recovery in potato plants depends on several factors such as cultivar type, weather, fertilizer rates and type, irrigation amounts and soil types, most researchers have reported a plant N recovery between 20 to70% of the applied N under non-limiting conditions of fertilizer and irrigation for potato production [55–59]. The plant N uptake values simulated by the model in this study were in agreement with the measured values reported in literature. The N that leached below the root zone was no longer available to the crops and may move to local springs and river. Nitrogen leaching loss depends on several factors such as weather, crop type, soil characteristics, topography, drainage intensity and management practices. Most studies, under different settings, have reported N leaching loss between 20 to 50% of the applied N. For example, Unlu et al. [58] reported 20% of the applied N was lost via leaching in sandy soil under potato production when 843 mm of irrigation water and 400 kg ha^-1^ N were applied. In another study on model simulations, Verhagen [60] showed that 30% of the applied N was lost via leaching from potato production on clay and loamy soils. Giletto and Echeverrı´a [61] reported mean N leaching loss of 12 to 57% from a Typic Argiudoll soils under potato production under hydric excess of 73 and 479 mm water, respectively. Estimated amounts of N lost via leaching in the present study were in agreement with the measured values reported by most researchers for potato production systems. Gaseous losses of N via denitrification and volatilization have been reported to be smaller in magnitude in well drained soils for potato production [62]. A study by Hyatt et al. [63] on irrigated potato grown on Entic Hapludoll soil, reported 0.25 to 0.49% of the applied N (as granular urea) was lost as nitrous oxide. Gaseous loss of N by via denitrification and volatilization has been found to depend on several factors (temperature, soil moisture and pH, soil type, fertilizer type, improper irrigation and drainage) and is highly variable over space and time 62]. Liu et al. [64] measured the gaseous N losses during a 2-year rotation with winter wheat and maize grown in a clay loam soil finding 4 to7% of the fertilizer N was lost via denitrification and volatilization. Nitrogen lost via denitrification and volatilization predicted in the current study were in agreement with the values reported by researchers in different cropping systems; nevertheless they represent a considerable area of uncertainty.

Leaching was a major loss pathway for N in this system. The source of N in leaching losses may originate from direct sources such as fertilizer (referred to as direct leaching), or from indirect sources such as mineralization of soil organic matter, organic amendments or left over plant residues (refereed as indirect leaching). In this study, fertilizer N contributed 79 to 82% towards the total input of N, whereas mineralization of soil organic matter and crop residue contributed 8 to 11% towards the total N input. Thus, the contribution of direct sources towards N leaching might be high in Florida sandy soils due to shallow root system of potato and poor N and water holding capacity of the sandy soil. Crop residue (roots and shoots) left in the field after the tuber harvest represented a significant amount of N (64 to 110 kg ha^-1^N) which could create potential for indirect N leaching upon their mineralization and in absence of a subsequent N recovery cover crop. Typically, two months of fallow period exits between harvest of tubers and planting of the fall row crop (silage corn) at the study site. The fallow period is characterized by summer rains which pose a threat to N released from rapidly mineralizing potato vines. Kraft and Stites [65] reported an average of 40 kg ha^-1^ plant residue N was left in the field after tuber harvest in the Wisconsin Central Sand Plain (WCSP) area that had the potential to mineralize rapidly and leach below the root zone even before utilization by a subsequent crop. In another study by Bundy and Andraski [39] on irrigated sandy soils in WCSP found that N left in the crop residue after harvest was not recovered in the subsequent crop and was lost by leaching.

Thus the study site contributes N to the underlying hydrosphere through direct leaching loss as well as creates a potential for indirect leaching from two main N sources 1) left over mineral N in the soil profile after crop harvest and, 2) N from mineralization of crop residue left in the field. The site contributed an average (± standard deviation) of 111 ± 18 kg ha^-1^ season^-1^N through leaching by the end of crop season and created potential for losing an average 73 ± 21 kg ha^-1^ season^-1^N from mineralization of crop residue and 19 ± 7 kg ha^-1^ season^-1^ N from mineral N left in soil profile after crop harvest. Further, this study site, which is marked by unconfined aquifers and eroded Hawthorne formation consisting of thin and pocketed clay mantle atop the limestone [30], makes the site vulnerable to fast loading rates of nitrate-N directly into groundwater via leaching. Hence BMPs should focus on controlling both the forms of N leaching losses (direct as well as indirect) from potato production in the MSRB. Direct leaching losses of N might occur due to poor timing or higher rates of N fertilizer application—an area that requires further investigation. Errebhi et al. [66] reported that direct leaching losses can be reduced by reducing the amounts of N applied at planting. Strategies to reduce nitrate leaching into groundwater from potato production in sandy soils have been reviewed by Shrestha et al. [67]. At present, there is no consensus or a BMP to manage the potato vines after application of desiccant to prevent indirect N leaching losses from the rapidly mineralizing potato vines.

Nitrogen leaching from the crop root zone and its subsequent loading to groundwater has been studied using several techniques. For example Kraft and Stites [65] used a novel “water year method” to estimate nitrate loading to groundwater. Meisinger and Randall [21] used simple mass balance to calculate long term potentially leachable N to groundwater. Sebilo et al., [68] used tracer technique to study the fate of isotopically labeled N fertilizers in a three—decade-long in situ tracer experiment and measured rates at which fertilizer-derived N was exported to the hydrosphere. In this study we presented a model based approach to predict the fates of N in an irrigated potato production system in sandy soil. These estimates can be used as surrogates for approximating the N loading rates to the environment and potential leachable N to estimate groundwater nitrate loading rates. Nitrogen lost through leaching after it leaves the root zone is considered an economic loss to farmers, an agronomic loss to plants and has environmental implication for the society. Further study is required to address the causes of N leaching from potato production in the MSRB and propose solutions to minimize the losses. We acknowledge that natural factors such as soil texture, heavy rainfall events or shallow root system of the potato plant cannot be altered, however management related factors of irrigation and N management must be given consideration to reduce environmental loading of N in the MSRB.

## Conclusion

This research explored the major sources and sinks of N for potato grown under center-pivot irrigated sandy soil in a karst dominated agricultural system. Model derived environmental N loading rates were in good agreement with the observed values for the study site. The model derived N budget indicated that leaching of N was the major loss associated with potato production in sandy soil and accounted 25 to 38% of the total seasonal N input. Nitrogen lost via leaching at the end of the crop season was substantial (111 ± 18 kg ha^-1^ season^-1^N). Further the crop residue left in the fields after tuber harvest represented a significant amount of N left in the soil (ranged between 64 to 110 kg ha^-1^N) and would create potential for losing an average of 73 ± 21 kg ha^-1^ season^-1^N resulting from mineralization of crop residue and in absence of a subsequent cover crop. Mineral N left in the soil after crop harvest amounted to 19 ± 7 kg ha^-1^ season^-1^ and represents an additional potential leachable N. A BMP must focus on controlling both direct and indirect leaching losses from such a production system. There is a need to investigate the causes of the N loss from such system and potential solutions to minimize them in order to preserve the water quality of the Suwannee River and associated springs.
